# Changes in corneal endothelial cells after trabeculectomy and EX-PRESS shunt: 2-year follow-up

**DOI:** 10.1186/s12886-018-0913-0

**Published:** 2018-09-10

**Authors:** Saki Omatsu, Kazuyuki Hirooka, Eri Nitta, Kaori Ukegawa

**Affiliations:** Department of Ophthalmology, Kagawa University Faculty of Medicine, 1750-1 Ikenobe, Miki, Kagawa 761-0793 Japan

**Keywords:** Trabeculectomy, EX-PRESS, Corneal endothelial cell density, Intraocular pressure

## Abstract

**Background:**

To compare trabeculectomy and EX-PRESS device implantation procedures for treating glaucoma and evaluate changes in corneal endothelial cell density (CECD).

**Methods:**

This study prospectively evaluated changes in the CECD in 60 eyes of 60 patients who underwent trabeculectomy and 50 eyes of 45 patients who underwent EX-PRESS device implantation. Baseline patient data recorded included age at surgery, sex, type of glaucoma medications, and lens status. Using a noncontact specular microscope, corneal specular microscopy was performed preoperatively at the central cornea and then at 6, 12, 18 and 24 months after surgery. CECD before and after surgery was compared using a paired *t*-test.

**Results:**

There was a significant decrease in the IOP and number of antiglaucoma medications in both groups after the surgery. The mean CECD in the trabeculectomy group was 2505 ± 280 cells/mm^2^ at baseline, while it was 2398 ± 274 cells/mm^2^ (*P* < 0.001), 2349 ± 323 cells/mm^2^ (*P* < 0.001), 2293 ± 325 cells/mm^2^ (*P* < 0.001), and 2277 ± 385 cells/mm^2^ (*P* = 0.003) at 6, 12, 18, and 24 months, respectively. However, the CECD in the EX-PRESS group was 2377 ± 389 cells/mm^2^ at baseline, while it was 2267 ± 409 cells/mm^2^ (*P* = 0.007), 2292 ± 452 cells/mm^2^ (*P* = 0.043), 2379 ± 375 cells/mm^2^ (*P* = 0.318), and 2317 ± 449 cells/mm^2^ (*P* = 0.274) at 6, 12, 18, and 24 months, respectively.

**Conclusions:**

As compared to trabeculectomy, EX-PRESS device implantation appears to be a safer procedure with regard to the endothelial cell loss risk.

## Background

The aim of glaucoma treatments is to slow disease progression while preserving visual functions without changing the patient’s quality of life. In general, these treatments are primarily designed to lower the intraocular pressure (IOP), with first approaches utilizing medical therapies with antiglaucomatous drugs. When maximal tolerable medical therapy is not able to sufficiently lower the IOP, patients are treated using trabeculectomy in order to prevent optic nerve damage or visual field deterioration. Although trabeculectomies are commonly used, the procedure is not without risk. Thus, the EX-PRESS drainage device (Alcon Laboratories, Fort Worth, TX) was designed and created as a safer alternative for controlling the IOP [1]. This device, which consists of a nonvalved stainless steel tube, is inserted under a partial-thickness scleral flap and serves as a connection between the anterior chamber and the subconjunctival space.

In young adults, the corneal endothelial cell density (CECD) is approximately 3000 cells/mm^2^. However, due to aging, the mean CECD value is reduced by 0.5 ± 0.6% every year [2]. This loss can be accelerated by several risk factors that include, surgery, argon laser iridotomy, and even glaucoma itself [3–9]. Although it has been previously shown that trabeculectomy can damage corneal endothelial cells [10–13], no changes have been observed in the CECD at either 1 or 3 months after EX-PRESS implantation surgery [12]. In contrast, Ishida et al. reported finding significant decreases in the CECD at 24 months after EX-PRESS implantation [14]. Even so, it should be noted that this previous study did not compare eyes undergoing implantation to a control group of eyes undergoing trabeculectomy.

The purpose of this study was to evaluate the long-term changes in corneal endothelial cells that occurred for up to 1 year after undergoing trabeculectomy and EX-PRESS shunt surgeries for the treatment of glaucoma.

## Methods

This observational study examined eyes undergoing treatments between April 2014 and May 2016 with either the EX-PRESS glaucoma filtration device or trabeculectomy. Trabeculectomies were performed in more than 200 eyes during the study observation period. Eyes selected for inclusion in the study were age and gender matched with the eyes from the EX-PRESS group. Written informed consent for participation in the study was obtained from participants. All procedures and follow-ups took place at the Kagawa University Hospital, Kagawa, Japan. When treatments included both eyes, data from the first eye operated on in the patient was selected and used for the study. The Institutional Review Board of the Kagawa University Faculty of Medicine approved this study protocol. In addition to the standard consent for surgery, all subjects provided written informed consent prior to their enrollment and taking part in the research study.

To be included in the study, patients were required to be older than 20 years of age, and have preoperative uncontrolled IOP despite being administered the maximum tolerated medical therapy. Exclusion criteria included having any significant ocular diseases or history in the operated eye (other than glaucoma or cataract), or exhibiting any other corneal epithelial or stromal disorders that could potentially cause issues during the specular microscopy. The study also excluded patients who did not provide specific reasons on why they were unable to complete the entire 1-year follow-up.

A noncontact specular microscope with an autofocus device, the Tomey EM-3000 (Tomey Corporation, Nagoya, Japan), was used for all observations. All measurements of the endothelial cell count were carried out at the center of the cornea, with the incorporated screen on the device used to visualize the endothelium. The device automatically measured the CECD, with cell density recorded as the number of cells per square millimeter. CECD measurements were obtained prior to and at 6 and 12 months after the surgery. Thereafter, all subsequent measurements were performed every 6 months.

Patients who had a history of vitreous surgery or severe vision loss of their fellow eye underwent the EX-PRESS glaucoma filtration device procedure, with all of the surgeries performed by one surgeon (KH). After administration of retrobulbar anesthesia with lidocaine 2%, all eyes were prepared and draped. In the first step of the procedure, after placing a corneal traction suture (5–0 silk suture), the surgeon dissected a fornix-based conjunctival flap, and then created a one-half thickness scleral flap (approximately 3.5×3.5 mm). Mitomycin C (MMC) was applied to the sclera over the proposed scleral flap site. Subsequently, after positioning 6 to 8 sponges containing 0.04% MMC solution in the subconjunctival space, the sponges were maintained in place for 3 to 5 min. Once the sponges were removed, the area was copiously irrigated using 250 ml of physiologic saline. After removing a block of clear cornea and trabecular meshwork tissue at the edge of the corneoscleral bed, peripheral iridectomy was performed, followed by suturing of the scleral flap using 6 to 7 monofilament 10–0 nylon sutures. Sutures were adjusted to ensure that a small amount of leakage could be observed around the scleral flap margin without causing any shallowing of the anterior chamber.

In the EX-PRESS group, a 26G needle was used to enter the anterior chamber slightly posterior to the blue-gray zone under the scleral flap, and the EX-PRESS (model P50) shunt was introduced into the anterior chamber through the needle track. Suturing of the scleral flap was performed using 2 to 4 monofilament 10–0 nylon sutures, while closure of the conjunctiva used 10–0 nylon sutures at the edges of the incision. For the conjunctiva, one or more of the horizontal mattress sutures were placed centrally. Once the anterior chamber was reformed by using a balanced salt solution, the wound was then checked for leaks. After instillation of a corticosteroid/antibiotic ointment, a sterile eye patch and shield was placed over the eye.

The procedure for the trabeculectomy group used a fornix-based, superior, one site approach, with the cataract surgery (phacoemulsification and intraocular lens insertion) combined with the trabeculectomy. The phacoemulsification procedure in the EX-PRESS group was performed via a 2.4 mm temporal clear cornea incision. After making the incision, the intraocular lens was then placed into the capsular bag. All patients were administered a topical corticosteroid (four times daily) and an antibiotic during the following 8 to 12 weeks after the surgery. If the filtration was judged to be too low by the surgeon or the IOP was too high to meet the target pressure, patients underwent suture lysis with an argon laser under topical anesthesia.

All statistical analyses were performed using SPSS version 19.0 (IBM, New York, NY). CECD and IOP were compared before and after surgery using paired *t*-tests. A *P* value less than 0.05 was considered to be statistically significant. Data are presented as the mean ± standard deviation.

## Results

Table 1 summarizes the patient characteristics. No significant differences were observed for the patient age, gender, and lens status between the trabeculectomy and EX-PRESS shunt groups. The number of combined cataract surgeries performed in the trabeculectomy and EX-PRESS shunt groups were 29 and 22, respectively (*P* = 0.72).

**Table 1:** Baseline patient characteristics of the trabeculectomy and EX-PRESS groups

	Trabeculectomy	EX-PRESS	*P* value
Mean Age (years)	63.7 ± 9.5	63.1 ± 12.3	0.85^a^
Gender (M/F)	31/33	30/20	0.22^b^
Diagnosis			
POAG	24	30	
NTG	24	12	
SG	11	8	
EG	5		
Lens status			0.06^b^
Phakia	47	28	
Pseudophakia	17	22	

*M* male, *F* female, *POAG* primary open-angle glaucoma, *NTG* normal-tension glaucoma, *SG* secondary glaucoma, *EG* exfoliation glaucoma

^a^independent t-test

^b^χ^2^ test

**Fig. 1:**
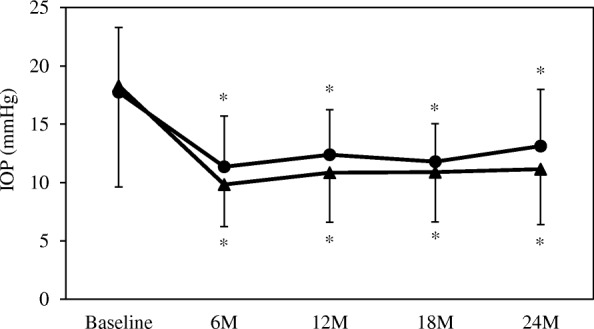
Mean intraocular pressure following trabeculectomy or treatment using the EX-PRESS glaucoma filtration device. The intraocular pressure was significantly reduced in both groups compared with baseline. *: *P* < 0.05 compared with baseline. ▲: trabeculectomy group, ●: EX-PRESS group

**Fig. 2:**
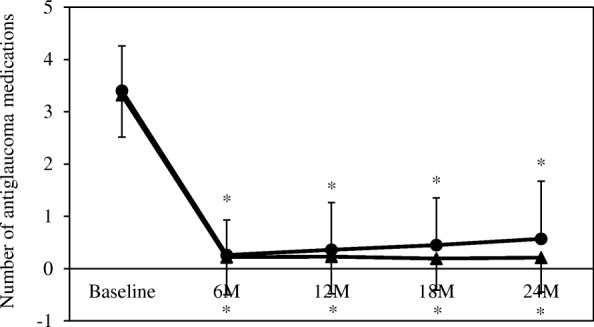
Mean antiglaucoma medications following trabeculectomy or treatment using the EX-PRESS glaucoma filtration device. The antiglaucoma medications were significantly reduced in both groups compared with baseline. *: *P* < 0.05 compared with baseline. ▲: trabeculectomy group, ●: EX-PRESS group

Significant decreases in the IOP and in the number of antiglaucoma medications were observed after the surgery in both procedures (Figs. 1 and 2). The mean IOP in the trabeculectomy group was 18.3 ± 8.7 mmHg at baseline, while it was 9.8 ± 3.6 mmHg, 10.9 ± 4.3 mmHg, 10.9 ± 4.3 mmHg, and 11.2 ± 4.8 mmHg at 6, 12, 18, and 24 months, respectively. The IOP in the EX-PRESS group was 17.7 ± 5.6 mmHg at baseline, while it was 11.4 ± 4.3 mmHg, 12.4 ± 3.9 mmHg, 11.8 ± 3.3 mmHg and 13.1 ± 4.8 mmHg at 6, 12, 18, and 24 months, respectively.

Table 2 shows the trends of the CECD over the course of the study between the two groups. The mean CECD in the trabeculectomy group was 2505 ± 280 cells/mm^2^ at baseline, while it was 2398 ± 275 cells/mm^2^, 2349 ± 323 cells/mm^2^, 2293 ± 325 cells/mm^2^, and 2277 ± 385 cells/mm^2^ at 6, 12, 18, and 24 months, respectively. The CECD in the EX-PRESS group was 2377 ± 389 cells/mm^2^ at baseline, while it was 2267 ± 409 cells/mm^2^, 2292 ± 452 cells/mm^2^, 2379 ± 375 cells/mm^2^, and 2317 ± 449 cells/mm^2^ at 6, 12, 18, and 24 months, respectively. In the trabeculectomy group, there was a significant reduction in the IOP from the baseline observed for all of the study visits. For the CECDs, however, at month 18 in the EX-PRESS group, there were no longer significant differences from the baseline noted for the density.

**Table 2:** Endothelial cell count before and after trabeculectomy and EX-PRESS

	Trabeculectomy (cells/mm^2^)	EX-PRESS (cells/mm^2^)
Baseline	2505 ± 280	2377 ± 389
6 months	2398 ± 275	2267 ± 409
*P* value	< 0.001	0.007
12 months	2349 ± 323	2292 ± 452
*P* value	< 0.001	0.043
18 months	2293 ± 325	2379 ± 375
*P* value	< 0.001	0.32
24 months	2277 ± 385	2317 ± 449
*P* value	0.003	0.27

Table 3 shows the pre- and postoperative CECD with or without cataract surgery. While there was a significant difference from baseline for the CECD at each of the study visits in the trabeculectomy group, the CECD in the EX-PRESS combined cataract surgery group at 12 months no longer exhibited any significant difference from the baseline. Furthermore, in the patients undergoing only the EX-PRESS procedure, the CECD did not exhibit any significant difference from baseline at any of the study visits.

**Table 3:** Endothelial cell count before and afyer trabeculectomy and EX-PRESS with or without cataract surgery

	Trabeculectomy (cells/mm^2^)		EX-PRESS (cells/mm^2^)	
	Combined cataract surgery	Trabeculectomy alone	Combined cataract surgery	EX-PRESS alone
Baseline	2539 ± 264 (*n* = 29)	2462 ± 281 (*n* = 35)	2512 ± 329 (*n* = 22)	2279 ± 415 (*n* = 28)
6 months	2429 ± 286 (*n* = 29)	2387 ± 253 (*n* = 35)	2309 ± 410 (*n* = 21)	2236 ± 413 (*n* = 27)
*P* value	< 0.001	0.018	0.034	0.11
12 months	2373 ± 344 (*n* = 29)	2338 ± 288 (*n* = 35)	2399 ± 333 (*n* = 19)	2216 ± 512 (*n* = 27)
*P* value	< 0.001	0.014	0.10	0.25
18 months	2344 ± 256 (*n* = 26)	2225 ± 393 (*n* = 27)	2429 ± 319 (*n* = 15)	2339 ± 419 (*n* = 19)
*P* value	< 0.001	0.008	0.29	0.81
24 months	2218 ± 393 (*n* = 10)	2272 ± 398 (*n* = 15)	2379 ± 416 (*n* = 8)	2284 ± 477 (*n* = 15)
*P* value	0.016	0.071	0.20	0.92

## Discussion

To ensure maintenance of the corneal integrity and transparency, the corneal endothelium is essential [15]. Aging, surgery, and trauma have been reported by several studies to be able to reduce the CECD [2, 4, 12–14]. Our current study showed that while there were no changes in the CECD after the EX-PRESS implantation, there were significant decreases observed in the CECD after the trabeculectomies.

Several studies that have investigated trabeculectomies reported finding a reduction in the postoperative CECD after the procedure [10, 11, 16]. While several possible mechanisms for the reductions in CECD after trabeculectomy have been proposed, the exact mechanism responsible for the endothelial cell loss after trabeculectomy has yet to be fully clarified and is most likely multifactorial. Although it has been shown that MMC has a toxic effect on the corneal endothelium [17], other trabeculectomy studies have reported finding a decrease in CECD without the use of MMC [16]. Therefore, this indicates that other factors might be contributing to the observed endothelial damage. Our study also showed that were no changes in the CECD after 18 months or 24 months in the EX-PRESS shunt implantation cases that were administered MMC during surgery. Therefore, our current results indirectly support the possibility that other factors might contribute to the endothelial damage.

This study also showed that there were changes in the CECD after 6 months or 12 months in the EX-PRESS shunt implantation cases. Since there was no effect on the CECD after the EX-PRESS shunt implantation without cataract surgery, this suggests that the cataract surgery may be responsible for these changes. Casini et al. also found that there were no changes in the CECD at 1 or 3 months after the EX-PRESS shunt implantation surgery [12]. In contrast, Ishida et al. showed that there was a significant decreased in the CECD at 24 months after the EX-PRESS shunt implantation [14]. The authors speculated that the reason for the differences seen in these studies is that the decreases in the CECD are only observed after a specific length of time. In the current study, however, during the initial 24 months after the EX-PRESS shunt implantation, we did not observe any decreases of CECD, with these changes occurring at 24 months after the implantation.

Although there were no changes in the CECD at 18 months or 24 months after the EX-PRESS shunt implantation, we did observe significant decreases at 6 or 12 months after the implantation. This suggests that the observed recovery of the CECD might be the result of cellular migration from the peripheral cornea.

Suturing of the scleral flap was performed using 6 to 7 10–0 nylon sutures in the trabeculectomy group and 2 to 4 10–0 nylon sutures in the EX-PRESS group. The physical properties of the apertures that connect the anterior chamber to the sub-scleral space are not same in the two procedures. Since the aqueous flow through EX-PRESS is probably different from the flow through sclerectomy of the trabeculectomy, the numbers of sutures were difference between the two techniques.

There were several limitations for our current study. First, it is possible that the area of the cornea examined might not have been the same at each of the visits. Second, the number of patients included in this study was quite small. Third, there was only a short follow-up period, which might not have been long enough to observe the changes. Furthermore, the EX-PRESS shunt consists of a stainless steel tube, and after insertion under a partial-thickness scleral flap, it provides a connection to the anterior chamber. Therefore, long-term follow-ups of patients will need to be performed in order to verify the effect of the EX-PRESS implantation on CECD. In addition, it should also be noted that we have combined data from several types of glaucoma, which could be an issue, as the pathogenesis could vary in each of the different types. There was a bias in the selection of patients. Patients who had a history of vitreous surgery or severe vision loss of their fellow eye underwent the EX-PRESS glaucoma filtration device procedure. As a result, the above limitations could potentially limit the general applicability of the current results. Additional studies that perform evaluations of the long-term effect of glaucoma surgery on CECD will need to be undertaken in the future.

## Conclusions

In conclusion, our comparison of the EX-PRESS shunt implantation and trabeculectomy procedures showed there were no effects on the corneal endothelial cells until at least 24 months after the initial surgery. These findings suggest the benefit of using this procedure in patients who have a lower CECD prior to the surgery.

## Abbreviations

CECD: Corneal endothelial cell density
IOP: Intraocular pressure
MMC: Mitomycin C
